# Biological Characterization of Commercial Recombinantly Expressed Immunomodulating Proteins Contaminated with Bacterial Products in the Year 2020: The SAA3 Case

**DOI:** 10.1155/2020/6087109

**Published:** 2020-07-06

**Authors:** Sara Abouelasrar Salama, Mirre De Bondt, Nele Berghmans, Mieke Gouwy, Vivian Louise Soares de Oliveira, Sergio C. Oliveira, Flavio A. Amaral, Paul Proost, Jo Van Damme, Sofie Struyf, Mieke De Buck

**Affiliations:** ^1^KU Leuven, Department of Microbiology and Immunology, Rega Institute, Laboratory of Molecular Immunology, Herestraat 49, Box 1042, 3000 Leuven, Belgium; ^2^Imunofarmacologia, Departamento de Bioquímica e Imunologia, Instituto de Ciências Biológicas, Universidade Federal de Minas Gerais, Av. Antonio Carlos 6627, Pampulha, Belo Horizonte, 31270-901 Minas Gerais, Brazil; ^3^Departamento de Bioquímica e Imunologia, Instituto de Ciências Biológicas, Universidade Federal de Minas Gerais, Av. Antonio Carlos 6627, Pampulha, Belo Horizonte, 31270-901 Minas Gerais, Brazil

## Abstract

The serum amyloid A (SAA) gene family is highly conserved and encodes acute phase proteins that are upregulated in response to inflammatory triggers. Over the years, a considerable amount of literature has been published attributing a wide range of biological effects to SAAs such as leukocyte recruitment, cytokine and chemokine expression and induction of matrix metalloproteinases. Furthermore, SAAs have also been linked to protumorigenic, proatherogenic and anti-inflammatory effects. Here, we investigated the biological effects conveyed by murine SAA3 (mu rSAA3) recombinantly expressed in *Escherichia coli*. We observed the upregulation of a number of chemokines including CCL2, CCL3, CXCL1, CXCL2, CXCL6 or CXCL8 following stimulation of monocytic, fibroblastoid and peritoneal cells with mu rSAA3. Furthermore, this SAA variant displayed potent *in vivo* recruitment of neutrophils through the activation of TLR4. However, a major problem associated with proteins derived from recombinant expression in bacteria is potential contamination with various bacterial products, such as lipopolysaccharide, lipoproteins and formylated peptides. This is of particular relevance in the case of SAA as there currently exists a discrepancy in biological activity between SAA derived from recombinant expression and that of an endogenous source, i.e. inflammatory plasma. Therefore, we subjected commercial recombinant mu rSAA3 to purification to homogeneity via reversed-phase high-performance liquid chromatography (RP-HPLC) and re-assessed its biological potential. RP-HPLC-purified mu rSAA3 did not induce chemokines and lacked *in vivo* neutrophil chemotactic activity, but retained the capacity to synergize with CXCL8 in the activation of neutrophils. In conclusion, experimental results obtained when using proteins recombinantly expressed in bacteria should always be interpreted with care.

## 1. Introduction

Contamination of medical drugs with bacterial products has been a constant concern long before recombinant technology was introduced. Lipopolysaccharide (LPS) from Gram-negative bacteria such as *Escherichia coli* (*E. coli*) is of particular relevance. Indeed, such endotoxins provoke fever and other inflammatory effects upon systemic or local injection. Scientifically, due to minor bacterial contamination of cell cultures or contamination with LPS during post-processing, the presence of minor LPS concentrations in natural cell culture-derived preparations of immunomodulators such as cytokines has significantly complicated their biological characterization [1]. For instance, interferon (IFN) produced *in vitro* in fibroblasts or leukocytes was found to be pyrogenic upon injection and this was long thought to be mediated by traces of LPS. However, following purification to homogeneity, IFN caused fever and could thus be considered as an endogenous pyrogen [2]. The need to eliminate LPS became increasingly evident in the seventies during the identification of endogenous pyrogens such as interleukin-1 (IL-1) [3]. It took four decades to isolate this inflammatory cytokine based on *in vivo* assays, due to biological interference with LPS [4, 5]. The fact that IFN was introduced for medical treatment forced the pharmaceutical industry to deal with LPS contamination [6, 7].

Concurrently, the introduction of recombinant DNA technology allowed the generation of vast quantities of cytokines through expression in *E. coli*. Nonetheless, this new biotechnology possesses the inherent risk of LPS contamination. Various studies have attempted to eliminate LPS and other bacterial products including formyl peptides and lipoproteins from preparations for clinical use by specific purification techniques, but complete elimination has proved to be challenging. Currently, four decades following the breakthrough of recombinant DNA technology, the scientific community is still suffering from this problem. Indeed, except for a few recognized biotechnology companies, a vast number of commercial entities offer recombinant proteins without paying full attention to the contamination of their products. Young researchers that have missed the emerging success of recombinant technology of the eighties are not aware of this problem and are inclined not to pay sufficient attention to this problem when purchasing expensive commercially available recombinant drugs.

In the present study, a member of the acute phase proteins, i.e., serum amyloid A (SAA), to which many inflammatory activities have been ascribed, has been re-evaluated. SAA consists of the variants SAA1, SAA2, SAA3 and SAA4 and is mainly expressed in the liver, but it can also be locally expressed in a variety of tissues and cell types. Whereas SAA1 and SAA2 serum levels increase up to 1000-fold during the acute phase response, SAA4 is constitutively expressed in the liver [8]. The *SAA3* gene is generally believed to be a pseudogene in humans [9], but in mice, SAA3 is the most abundantly extrahepatically expressed SAA variant [10]. Murine SAA3 is chemotactic for macrophages and Lewis lung carcinoma (LLC) cells [11, 12]. SAA3 originating from hypertrophic 3T3-L1 murine adipocytes also stimulates the migration of these cells in concert with the chemokine CCL2 [13]. Moreover, it has been shown that murine SAA3 recombinantly expressed in *E. coli* (mu rSAA3) induces tumor necrosis factor-*α* (TNF-*α*) mRNA in peritoneal macrophages and murine colonic CMT-93 cells [14, 15] and matrix metalloproteinases (MMPs) and CCL5 mRNA in murine adenocarcinoma VMR cells [16]. In addition, protumorigenic and proatherogenic properties have been assigned to murine SAA3 [12, 16–18]. Experiments using SAA3 knockout mice revealed that SAA3 induces pathogenic Th17 cells [19] and contributes to podocyte-derived inflammation and consequent kidney damage in a mouse model of type I diabetes [20]. On the other hand, it has been suggested that mu rSAA3 relays an antibacterial effect in the colon through the induction of mucin 2 [15, 21]. Such an anti-inflammatory effect was also shown in SAA3 knockout mice, which displayed a higher secretion of proinflammatory cytokines and chemokines upon stimulation with inflammatory agents and a worsened outcome of the disease model compared to wildtype mice [22–24]. Here, the activity of commercially available mu rSAA3 produced by different companies was investigated. It was found that such SAA3 preparations could exert multiple inflammatory activities known to be mediated by a spectrum of cellular receptors including TLRs. However, upon further purification to homogeneity via reversed-phase high-performance liquid chromatography (RP-HPLC), mu rSAA3 was found to be devoid of most of the TLR-mediated inflammatory effects observed with its untreated counterpart. As such, caution must be taken when testing commercially available proteins, particularly those derived from recombinant technology, due to contamination with impurities. Hence, purification to homogeneity should be considered before usage.

## 2. Materials and Methods

### 2.1. Reagents

Recombinant human SAA1 (hu rSAA1; 300-53), recombinant human CXCL8 (200-08M), recombinant human IL-1*β* (200-01B) and recombinant human TNF-*α* (300-01A) were purchased from PeproTech (Rocky Hill, NJ, USA). Recombinant murine SAA3 (mu rSAA3) was purchased from either MyBioSource (MBS1043052; San Diego, CA, USA) or Gentaur (CSB-EP361411Moe1; Kampenhout, Belgium). Lipopolysaccharide (LPS; 0111:B4) derived from *E. coli* and peptidoglycan (PGN; 77140) derived from *Staphylococcus aureus* were purchased from Sigma-Aldrich (St. Louis, MO, USA). TAK-242 (HY-11109) was purchased from MedChemExpress (Monmouth Junction, NJ, USA) and was solubilized in DMSO.

### 2.2. Cell Cultures

Human CD14^+^ monocytes were purified from one-day-old buffy coats, derived from healthy donors (Belgian Red Cross, Mechelen, Belgium), through density gradient centrifugation and positive selection (MACS, Miltenyi Biotec, Bergisch Gladbach, Germany) as previously described [25]. Human neutrophils were isolated from fresh blood derived from healthy donors via density gradient centrifugation as previously described [26].

The murine macrophage cell line RAW264.7 [American Type Culture Collection (ATCC), Manassas, VA, USA] was grown in Dulbecco's modified Eagle's medium (DMEM; Lonza, Verviers, Belgium) supplemented with glucose (4.5 g/l), 1 mM sodium pyruvate and 10% fetal calf serum (FCS; Gibco, Thermo Fisher Scientific, Waltham, MA, USA). The murine fibroblast cell line L929 (ATCC) was grown in minimum essential medium (MEM) Rega (Thermo Fisher Scientific) supplemented with 10% FCS. Murine LLC cells (ATCC) were grown in DMEM supplemented with glucose (4.5 g/l) and 10% FCS. Murine peritoneal cells were extracted from healthy female NMRI mice (Charles River, Wilmington, MA, USA) that were kept in a specific pathogen-free environment. After euthanizing the mice, peritoneal lavages were carried out with 5 ml of phosphate-buffered saline (PBS) supplemented with 20 U/ml of heparin (LEO Pharma, Lier, Belgium) and 2% FCS.

### 2.3. Induction Experiments

CD14^+^ monocytes were seeded in 48-well plates (2 × 10^6^ cells/ml, 500 *μ*l/well) in Roswell Park Memorial Institute- (RPMI-) 1640 medium (Lonza) supplemented with 0.5% human serum albumin (HSA; Belgian Red Cross, Brussels, Belgium) and stimulated for a period of 24 h. RAW264.7 cells were seeded in a 48-well plate (90 × 10^4^ cells/ml, 500 *μ*l/well) in culture medium. After 48 h, culture medium was replaced with DMEM supplemented with glucose (4.5 g/l), 1 mM sodium pyruvate and 0.5% HSA in which cells were stimulated for a period of 24 h. L929 cells were seeded in a 48-well (for chemokine induction) or a 6-well (for mRNA induction) plate (30 × 10^4^ cells/ml, 500 *μ*l or 2 ml/well, respectively) in culture medium. After 48 h, culture medium was replaced with MEM Rega supplemented with 0.5% HSA in which cells were stimulated for 24 h for chemokine induction or for 16 h for mRNA induction. LLC cells were seeded in 6-well plates (20 × 10^4^ cells/ml, 2 ml). Following 3 days, cell culture medium was replaced with fresh medium (1.2 ml) and cells were stimulated for a period of 16 h. Peritoneal lavages from different mice were pooled, seeded in 48-well plates (500 *μ*l/well) and stimulated for a period of 24 h. All induction experiments were carried out at 37°C and 5% CO_2_. In the case of chemokine protein measurement, cell supernatants were collected and stored at -20°C until chemokine quantification.

### 2.4. mRNA Extraction and Quantitative Real-Time Polymerase Chain Reaction (qRT-PCR)

mRNA was extracted from stimulated LLC cells and L929 cells using an RNeasy Mini Kit (74106; Qiagen, Hilden, Germany) following the manufacturer's instructions. *β*-Mercaptoethanol was added to the RLT lysis buffer. After determining the mRNA concentration, purity and quality via a NanoDrop 3300 Fluorospectrometer (Thermo Fisher Scientific), single-stranded cDNA was produced from the extracted mRNA via a High-Capacity cDNA Reverse Transcription Kit (4368814; Thermo Fisher Scientific) according to the manufacturer's instructions. Reverse transcription was initiated in a GeneAmp PCR System 9700 thermal cycler (Applied Biosystems, Foster City, CA, USA) using the following program: 10 min at 25°C for primer annealing, 2 h at 37°C for reverse transcription and 5 min at 85°C to end the reaction. Samples were stored at -20°C until determination of gene expression. Quantitative real-time polymerase chain reaction (qRT-PCR) was performed in 96-well MicroAmp Fast plates (Thermo Fisher Scientific). The following commercial primer/probe mixes were used: mu SAA1 (Mm.PT.58.21905888; exon location 2-3; IDT, Leuven, Belgium), mu SAA3 (Mm.PT.58.32060531; exon location 1-3; IDT), mu CCL2 (Mm.PT.58.42151692; exon location 1-3; IDT) and mu Tbp (housekeeping gene; Mm.PT.39a.22214839; exon location 4-5; IDT). A master mix solution consisting of 15 *μ*l TaqMan Gene Expression Master Mix (Thermo Fisher Scientific), 1.5 *μ*l primer mix and 3.5 *μ*l RNase-free water per sample was mixed with 10 *μ*l sample containing cDNA. Fifty ng of cDNA was used for each reaction and each sample was tested in duplicate. Reverse transcriptase-negative samples from the conversion to cDNA were included to exclude genomic DNA contamination. qRT-PCR was performed in a 7500 FAST Real-Time PCR thermal cycler (Thermo Fisher Scientific) using the following program: 2 min at 50°C, 10 min at 95°C and 45 cycles of 15 sec at 95°C and 1 min at 60°C. Data were normalized against the expression of mu Tbp and against normalized values originating from control cells using the 2^−ΔΔCt^ method [27].

### 2.5. Enzyme-Linked Immunosorbent Assay (ELISA)

Quantification of chemokines in cell supernatants was done by ELISA. The human CXCL8 ELISA was developed in our laboratory using monoclonal mouse anti-human CXCL8 (MAB208) and biotinylated polyclonal goat anti-human CXCL8 (BAF208) antibodies purchased from R&D Systems (Minneapolis, MN, USA) [26]. Similarly, the human CCL2 ELISA was also developed in our laboratory with reagents from R&D Systems [monoclonal mouse anti-human CCL2 (MAB679) and biotinylated monoclonal mouse anti-human CCL2 (BAF279)] [28]. Human CCL3 and murine CCL2, CXCL1 and CXCL6 were measured with a specific Duoset ELISA kit following the manufacturer's instructions (R&D Systems).

### 2.6. Neutrophilic Granulocyte Activation and Migration Assays

Neutrophil migration was determined in a 48-well Boyden Microchamber Assay (Neuro Probe, Gaithersburg, MD, USA) as described [26]. The chemotactic index (CI) was calculated by dividing the number of cells migrated to the chemoattractant by the number of cells migrated to buffer alone. The neutrophil shape change assay was carried out as described [26]. Synergy was defined as a response to the combination of two chemoattractants that exceeded the sum of the responses obtained for the chemoattractants alone. The ethical committee of University Hospitals (UZ) Leuven approved experiments involving human neutrophils (project S58418).

### 2.7. *In Vivo* Cell Recruitment

The *in vivo* chemotactic potential of mu rSAA3 after intra-articular (i.a.) injection was determined in C57BL/6J male mice (8-10 weeks old; Centro de Bioterismo of the Universidade Federal de Minas Gerais). Mice were kept in a conventional housing facility and received food and water *ad libitum*. The mice were first anaesthetized through intraperitoneal (i.p.) injection of a mixture of 3.75% (*w*/*v*) of ketamine (Syntec, Santana de Parnaíba, Brazil) and 0.25% (*w*/*v*) of xylazine (Syntec) diluted in PBS. Afterwards, the mice were injected i.a. in the knee with 10 *μ*l of stimulus in one joint and, as a control, the other joint was injected with 10 *μ*l of 0.9% sodium chloride. After 3 h, mice were euthanized by an i.p. injection of a ketamine/xylazine overdose. Knee joints were washed twice with 5 *μ*l of PBS supplemented with 3% bovine serum albumin (BSA) and cytospins were prepared for differential cell counts. After drying, cells on the glass slides were stained with Panoptic Solutions (Laborclin, PR, Brazil). The slides were evaluated microscopically (500x magnification) by two individuals independently. All procedures were approved by the local animal ethics committee of the Federal University of Minas Gerais (295/2018).

The i.p. injections were carried out on female NMRI mice kept in a specific pathogen-free environment (7-8 weeks old; Charles River). Mice were injected with either PBS or vehicle, as control, or the stimulus of interest (100 *μ*l/mouse). Following a 2 h incubation period, mice were euthanized through subcutaneous injection of Dolethal (Vetoquinol, Northamptonshire, UK; 500 *μ*l/mouse), followed by cervical dislocation. The abdominal cavity was washed with 5 ml of PBS supplemented with 20 U/ml heparin and 2% FCS during 1 min. The percentage of neutrophils in the lavages was determined through either flow cytometry or cytospins. Cytospins were stained with Hemacolor Solutions (Merck, Darmstadt, Germany) and counted independently by two individuals. All procedures were approved by the local ethics committee for animal experiments (P070/2015; KU Leuven).

### 2.8. Flow Cytometry

Murine peritoneal cell suspensions were diluted to 5 × 10^5^ cells/ml in fluorescence-activated cell sorting (FACS) buffer (PBS with 2% FCS and 2 mM ethylenediaminetetraacetic acid). To exclude dead cells from the analysis, cells were incubated with Zombie Aqua viability dye (BioLegend, San Diego, CA, USA) for 15 min at room temperature (RT). Afterwards, the cells were washed with FACS buffer. To block the Fc receptors, cell suspensions were incubated with Fc receptor blocking agent (anti-CD16/CD32; Miltenyi Biotec) for 15 min at RT. Afterwards, the cells were washed and stained with the following anti-mouse antibodies for 30 min at 4°C: APC-labeled anti-CD11b (clone M1/70; eBioscience, Thermo Fisher Scientific) and BUV395-labeled anti-Ly-6G (clone 1A8; BD Biosciences, San Jose, CA, USA). The cells were subsequently washed and fixed with 0.4% formaldehyde in PBS. Acquisition was carried out using an LSRFortessa X-20 cell analyzer (BD Biosciences) and data analysis was carried out using FlowJo software (Tree Star, Ashland, OR, USA).

### 2.9. Reversed-Phase High-Performance Liquid Chromatography (RP-HPLC) and Mass Spectrometry (MS)

To purify mu rSAA3, RP-HPLC (Waters 600 HPLC System) was utilized. Mu rSAA was purified using a C8 Aquapore RP-300 HPLC column (220 × 2.1 mm; PerkinElmer, Norwalk, CT, USA). The loading solvent was composed of 0.1% trifluoroacetic acid (TFA). Following the loading of mu rSAA3 onto the column, elution was achieved by a gradually increasing acetonitrile (ACN) gradient. UV absorbance measured at 214 nm reflected protein concentration. Following chromatographic separation, RP-HPLC fractions were diluted 1 : 10 in 0.1% TFA and manually injected into a mass spectrometer (AmaZon SL, Bruker Daltonics, Bremen, Germany) to evaluate their purity. Pure fractions were pooled following MS characterization and lyophilized. Lyophilized material was dissolved in PBS and utilized for biological assays.

### 2.10. Limulus Amoebocyte Lysate (LAL) Assay

The LAL assay was performed using a specific kit following the manufacturer's instructions (bioMérieux Marcy-l'Étoile, France).  Table 1 shows the endotoxin level in impure and RP-HPLC-purified mu rSAA3 and hu rSAA1.

### 2.11. Statistical Analysis

The data were initially analyzed using the Kruskal-Wallis test for comparison of multiple groups. Afterwards, pairwise comparison was performed using the Mann-Whitney *U* test. Statistical analysis was performed using Statistica 13.5 software (StatSoft, Dell, Aliso Viejo, CA, USA). Statistical significance was established at a *p*-value of less than 0.05.

## 3. Results

### 3.1. Inflammatory Mediators Upregulate SAA3 Expression in Murine Lewis Lung Carcinoma Cells

**Figure 1:**
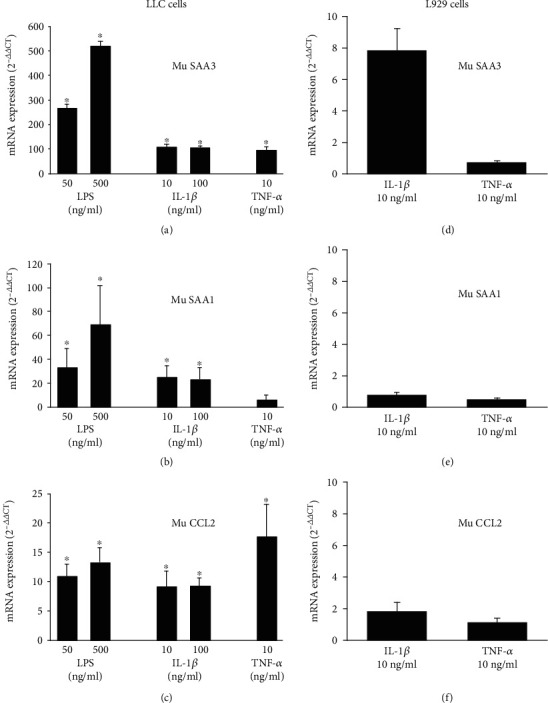
Inflammatory mediators stimulate the expression of mu SAA1, SAA3 and CCL2 mRNA in murine Lewis lung carcinoma cells (LLC cells) and the expression of SAA3 mRNA in murine L929 fibroblasts. LLC cells (a–c) or L929 cells (d–f) were stimulated with LPS (50 or 500 ng/ml; a–c), IL-1*β* (10 ng/ml; a–f), IL-1*β* (100 ng/ml; a–c), TNF-*α* (10 ng/ml; a–f) or were left untreated. After 16 h, total cell RNA was extracted and single-stranded cDNA was produced. Expression of mu SAA3 (a and d), mu SAA1 (b and e) and mu CCL2 (c and f) mRNA was detected via real-time polymerase chain reaction (RT-PCR) and upregulation of the expression was determined using the 2^−ΔΔCt^ method. Data represent the mean fold mRNA change ± SEM in stimulated cells compared to untreated cells and are derived from 3 (a–c) or 2-8 (d–f) independent experiments. Statistically significant upregulation of mRNA compared to control cells, determined by the Mann-Whitney *U* test, is indicated by asterisks (^∗^*p* < 0.05).

SAA3 expression is upregulated in several mouse models (e.g., diabetes and obesity), although its regulatory mechanisms have been studied poorly [12, 29–32]. Therefore, LLC cells were stimulated for a period of 16 h with different concentrations of the inflammatory mediators LPS, IL-1*β* or TNF-*α*, whereafter SAA3, SAA1 and CCL2 mRNA were determined by qRT-PCR (Figures 1(a)–1(c)). In comparison to control, LPS, IL-1*β* and TNF-*α* upregulated mRNA expression of SAA3 up to 521 ± 19-fold (LPS at 500 ng/ml), 108 ± 15-fold (IL-1*β* at 10 ng/ml), and 93 ± 16-fold (TNF-*α* at 10 ng/ml), respectively (*n* = 3; *p* < 0.05) (Figure 1(a)). Similarly, SAA1 mRNA was highly induced by LPS (up to 70 ± 32-fold upon stimulation with 500 ng/ml LPS; *n* = 3; *p* < 0.05), whereas this transcript was upregulated to a smaller extent when LLC cells were treated with IL-1*β*, reaching a maximal upregulation of the SAA1 gene of 25 ± 9-fold at 10 ng/ml of IL-1*β* (*n* = 3; *p* < 0.05) (Figure 1(b)). As a control, mRNA of the inflammatory chemokine CCL2 was also induced in LLC cells stimulated with LPS (up to 13 ± 2-fold at 500 ng/ml), IL-1*β* (up to 9 ± 3-fold at 10 ng/ml) or TNF-*α* (18 ± 6-fold at 10 ng/ml) (*n* = 3; *p* < 0.05) (Figure 1(c)). Expression of SAA3, SAA1 and CCL2 mRNA was also measured in murine L929 fibroblasts stimulated with IL-1*β* (10 ng/ml) or TNF-*α* (10 ng/ml) (Figures 1(d)–1(f)). Compared to unstimulated control cells, the SAA3 mRNA production was 7.81 ± 1.42-fold in IL-1*β*-stimulated cells (*n* = 8; *p* < 0.05) and 0.70 ± 0.13-fold in cells treated with TNF-*α* (*n* = 2). In contrast, the *SAA1* and *CCL2* genes were not upregulated in L929 cells treated with the same concentration of IL-1*β* or TNF-*α*.

### 3.2. Impure Murine Recombinant SAA3 Induces Chemokines in a Variety of Human and Murine Cell Types

**Figure 2:**
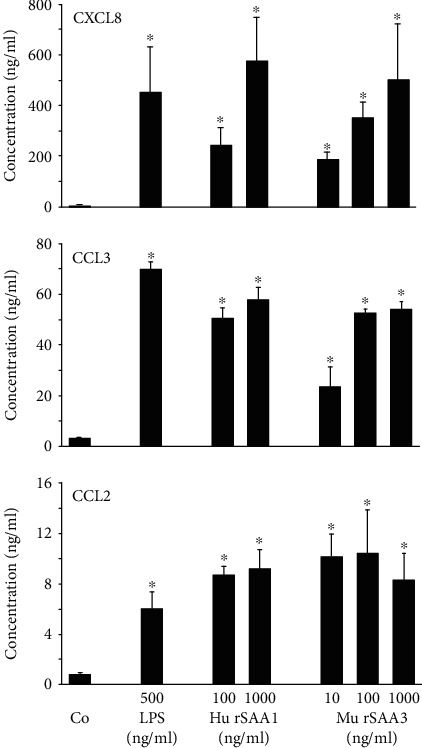
Impure mu rSAA3 induces chemokines in human CD14^+^ monocytes. Human CD14^+^ monocytes derived from buffy coats of healthy individuals were stimulated with LPS (500 ng/ml), hu rSAA1 (100 or 1000 ng/ml), mu rSAA3 (10-1000 ng/ml) or were left untreated (Co). After 24 h, supernatants were collected and levels of CXCL8, CCL3 and CCL2 were determined via specific ELISAs. Data represent the mean production of chemokine ± SEM derived from 4 independent experiments. Statistically significant differences from untreated control cells, determined by the Mann-Whitney *U* test, are indicated by asterisks (^∗^*p* < 0.05).

Next, we studied the potency of murine recombinant SAA3 (mu rSAA3; MyBiosource) to induce chemokines in human CD14^+^ monocytes and murine RAW264.7, L929 and peritoneal cells. Human CD14^+^ monocytes were stimulated with LPS (500 ng/ml), human recombinant SAA1 (hu rSAA1; 100 or 1000 ng/ml) or mu rSAA3 (10-1000 ng/ml). After 24 h, CXCL8, CCL3 and CCL2 levels were determined using specific ELISAs developed in our laboratory (Figure 2). Mu rSAA3 and hu rSAA1 induced CXCL8, CCL3 and CCL2 in a dose-dependent manner. Equal chemokine levels were produced by the monocytes upon stimulation with both SAA proteins or LPS, the maximal chemokine production being 576 ± 182,502 ± 223 and 453 ± 184 ng/ml of CXCL8 (Figure 2(a)),58 ± 5, 54 ± 4 and 69 ± 3 ng/ml of CCL3 (Figure 2(b)) and 9.2 ± 1.5, 8.3 ± 2.1 and 6.1 ± 1.3 ng/ml of CCL2 (Figure 2(c)) for hu rSAA1, mu rSAA3 (both at 1000 ng/ml) and LPS (500 ng/ml), respectively (*n* = 4; *p* < 0.05).

**Figure 3:**
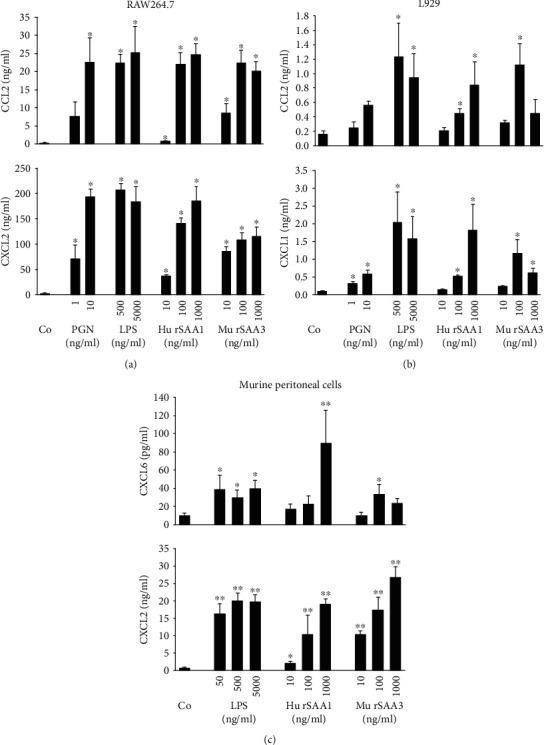
Impure mu rSAA3 induces chemokines in murine RAW264.7 macrophages, L929 fibroblasts and peritoneal cells. Murine RAW264.7 macrophages (a), murine L929 fibroblasts (b) and murine peritoneal cells derived from healthy mice (c) were stimulated with different concentrations of PGN (1 or 10 ng/ml; a and b), LPS (500 or 5000 ng/ml (a and b) or 50-5000 ng/ml (c)), hu rSAA1 (10-1000 ng/ml), mu rSAA3 (10-1000 ng/ml) or were left untreated (Co). After 24 h, supernatants were collected and the production of CCL2 (a and b), CXCL1 (b), CXCL2 (a and c) and CXCL6 (c) was determined via specific ELISAs. Data represent the mean production of chemokine ± SEM derived from 4 to 5 (a and b) or 6 (c) independent experiments. Statistically significant differences from untreated control cells, determined by the Mann-Whitney *U* test, are indicated by asterisks (^∗^*p* < 0.05; ^∗∗^*p* < 0.01).

Murine RAW264.7 macrophages and L929 fibroblasts were treated with different concentrations of PGN (1 or 10 ng/ml), LPS (500 or 5000 ng/ml), hu rSAA1 (10-1000 ng/ml) or mu rSAA3 (10-1000 ng/ml) for a period of 24 h. CCL2, CXCL1, CXCL2 and CXCL6 levels in supernatants were determined using specific sandwich ELISAs (Figures 3(a) and 3(b)). RAW264.7 cells produced statistically significant amounts of CCL2 and CXCL2 (Figure 3(a)), but not of CXCL1 and CXCL6 (data not shown). Again, similar chemokine levels were induced by the different stimuli. The maximal CCL2 production was 24 ± 4 (*p* < 0.05), 20 ± 3 (*p* < 0.05), 22 ± 7 (*p* < 0.05) and 25 ± 8 ng/ml (*p* < 0.05) upon stimulation of the macrophages with hu rSAA1, mu rSAA3 (both at 1000 ng/ml), PGN (10 ng/ml) or LPS (5000 ng/ml), respectively (*n* = 4‐5) (Figure 3(a), upper panel). Higher levels of CXCL2 were produced by these cells, the maximal chemokine production being 185 ± 28, 115 ± 21, 193 ± 16, and 207 ± 14 ng/ml when cells were treated with hu rSAA1, mu rSAA3 (both at 1000 ng/ml), PGN (10 ng/ml) or LPS (500 ng/ml), respectively (*n* = 4‐5; *p* < 0.05) (Figure 3(a), lower panel). L929 fibroblasts produced low, but statistically significant amounts of CCL2 and CXCL1 (Figure 3(b)), but not of CXCL2 or CXCL6 (data not shown). The maximal CCL2 production was 0.8 ± 0.3, 1.1 ± 0.3, 0.6 ± 0.1 (*p* = 0.06) and 1.2 ± 0.5 ng/ml, whereas that of CXCL1 was 1.8 ± 0.8, 1.1 ± 0.4, 0.6 ± 0.2 and 2.0 ± 0.9 ng/ml upon stimulation of the cells with hu rSAA1 (1000 ng/ml), mu rSAA3 (100 ng/ml), PGN (10 ng/ml) or LPS (500 ng/ml), respectively (*n* = 4‐5; *p* < 0.05) (Figure 3(b)).

To extrapolate the SAA3-induced production of chemokines to a more physiological setting, we also performed *ex vivo* induction experiments on murine peritoneal cells. Therefore, peritoneal lavages of untreated mice were incubated with different concentrations of LPS (50-5000 ng/ml), hu rSAA1 or mu rSAA3 (both 10-1000 ng/ml) during 24 h. CXCL2 and CXCL6 levels in supernatants were measured using specific ELISAs (Figure 3(c)). Maximal CXCL6 concentrations of 89 ± 36 pg/ml, 33 ± 118 pg/ml and 39 ± 10 pg/ml were reached at 1000 ng/ml of hu rSAA1, 100 ng/ml of mu rSAA3 and 5000 ng/ml of LPS, respectively (*n* = 6; *p* < 0.05) (Figure 3(c), upper panel). In addition, a dose-dependent induction of CXCL2 in the peritoneal cells was observed, with a maximal chemokine production of 19 ± 2, 27 ± 3 and 20 ± 2 ng/ml upon stimulation of cells with hu rSAA1, mu rSAA3 (both at 1000 ng/ml) or LPS (500 ng/ml), respectively (*n* = 6; *p* < 0.01) (Figure 3(c), lower panel).

### 3.3. Impure Mu rSAA3 Is a Potent Chemoattractant for Neutrophils *In Vitro* and *In Vivo*

**Figure 4:**
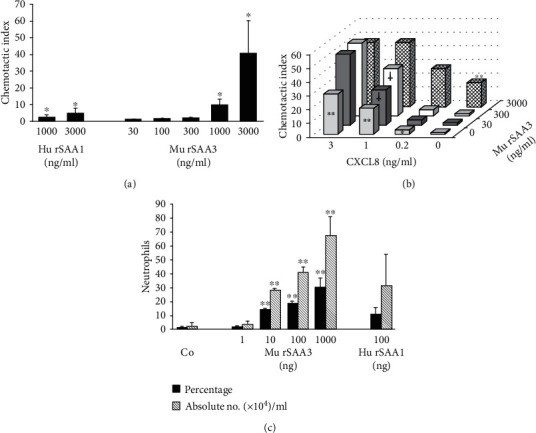
Impure mu rSAA3 is a potent neutrophil chemoattractant *in vitro* and *in vivo* and synergizes with CXCL8 in neutrophil migration. (a and b) The chemotactic activity of hu rSAA1 (1000 or 3000 ng/ml) and mu rSAA3 (30-3000 ng/ml) on human neutrophils was evaluated in the Boyden Microchamber Assay. The lower compartment of the microchamber was filled with chemoattractant alone (a) or with a combination of mu rSAA3 (30-3000 ng/ml) and CXCL8 (0.2-3 ng/ml) to assess synergy (b). The chemotactic potency is expressed as chemotactic index and is shown with (a) or without (b) SEM for 5-6 independent experiments. Statistically significant migration (compared to controls) and statistically significant synergy (compared to the sum of the values when both chemoattractants are tested separately), determined by the Mann-Whitney *U* test, are indicated by asterisks (^∗^*p* < 0.05; ^∗∗^*p* < 0.01) and daggers (^†^*p* < 0.05), respectively. (c) Female NMRI mice were injected i.p. with 100 *μ*l of mu rSAA3 (1-1000 ng), hu rSAA1 (100 ng) or PBS (Co; 2-8 mice per group). After 2 h, mice were sacrificed and peritoneal lavages were performed. Total cell counts in peritoneal lavages were determined and cytospins were prepared for differential leukocyte counts by 2 individuals independently. Data represent the mean percentage (black bars) or absolute number × 10^4^/ml (hatched bars) of neutrophils ± SEM derived from 2 to 3 independent experiments. Statistically significant recruitment of neutrophils compared to PBS-injected mice, determined by the Mann-Whitney *U* test, is indicated by asterisks (^∗∗^*p* < 0.01).

Murine SAA3 has previously been shown to be a weak chemoattractant of monocytic THP-1 cells and macrophages [11, 13]. To investigate whether commercial rSAA3 protein (MyBiosource) also stimulates the migration of neutrophils, as described for hu rSAA1 [26, 33, 34], Boyden chamber chemotaxis experiments were performed using human neutrophils (Figure 4(a)). Mu rSAA3 provoked a strong chemotactic response of neutrophils from 1000 ng/ml onwards, reaching a maximal CI of 41.1 ± 19.4 at 3000 ng/ml (*n* = 5; *p* < 0.05). In contrast, hu rSAA1 at 3000 ng/ml weakly stimulated neutrophil migration (CI = 5.1 ± 3.1; *n* = 5; *p* < 0.05). As a control, the established neutrophil chemoattractant CXCL8 strongly stimulated the migration of neutrophils (CI = 90.9 ± 66.5 at 3 ng/ml; *n* = 5; *p* < 0.05).

Since hu rSAA1 synergizes with CXCL8 in neutrophil chemotaxis [26], we also assessed the synergizing potency of mu rSAA3 in this setting (Figure 4(b)). Mu rSAA3 at 30 (CI = 1.4 ± 0.7; *n* = 6) and 300 ng/ml (CI = 1.4 ± 0.6; *n* = 6) synergized with CXCL8 at 1 ng/ml (CI = 19.0 ± 1.7; *n* = 6) reaching a CI of 26.3 ± 4.5 (*n* = 6; *p* < 0.05) and 34.7 ± 5.3 (*n* = 5; *p* < 0.05), respectively. Again, mu rSAA3 at 3000 ng/ml used as a single stimulus potently stimulated the migration of neutrophils (CI = 17.4 ± 3.6; *n* = 6; *p* < 0.01).

To test the chemotactic capacity of SAA3 *in vivo*, NMRI mice were injected i.p. with PBS, hu rSAA1 (100 ng) or different doses of mu rSAA3 (1-1000 ng). After 2 h, the percentage and absolute number of neutrophils in the peritoneal lavages (5 ml) were determined by differential leukocyte counting of cytospins (Figure 4(c)). Similarly as in the *in vitro* experiments, mu rSAA3 provoked a robust recruitment of neutrophils towards the peritoneal cavity. Already at a dose of 10 ng of mu rSAA3, 14.2 ± 0.6% of the cells in the peritoneal lavages displayed neutrophilic morphology (*n* = 5; *p* < 0.01 versus 1.3 ± 0.8% neutrophils in PBS-treated mice), corresponding to 28.26 ± 0.92 × 10^4^ neutrophils/ml (*p* < 0.01 versus 2.64 ± 1.61 × 10^4^ neutrophils/ml in PBS-treated mice). The chemotactic response of neutrophils towards SAA3 was dose-dependent and at 1000 ng of mu rSAA3, a maximal neutrophil recruitment of 29.9 ± 6.9% or 67.52 ± 12.99 × 10^4^/ml was reached (*n* = 6; *p* < 0.01). As already shown previously [25], hu rSAA1 (100 ng) also induced *in vivo* neutrophil recruitment, 11.0 ± 4.9% neutrophils being present in the peritoneal lavages, corresponding to 31.71 ± 21.71 × 10^4^ neutrophils/ml (*n* = 2).

### 3.4. The *In Vivo* Chemotactic Potency of Impure Mu rSAA3 Is Mediated through Activation of TLR4

**Figure 5:**
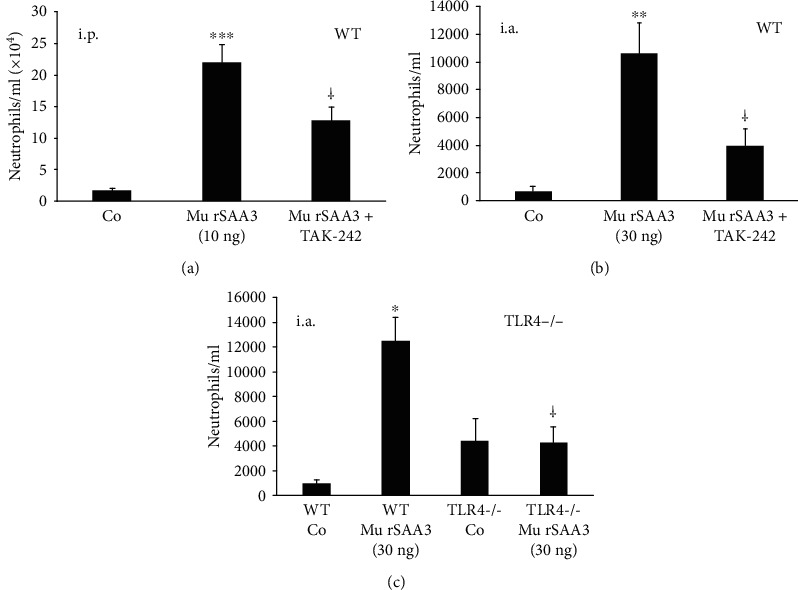
*In vivo* recruitment of neutrophils towards impure mu rSAA3 is mediated by activation of TLR4. (a) Female NMRI mice were injected i.p. with PBS (Co), mu rSAA3 (10 ng) or with a combination of mu rSAA3 (10 ng) and the TLR4 inhibitor TAK-242 (75 *μ*g; 9-12 mice per group). After 2 h, mice were sacrificed and peritoneal lavages were performed. (b) Male C57BL/6J mice were injected i.a. in the knee with 0.9% NaCl (Co), mu rSAA3 (30 ng), or with a combination of mu rSAA3 (30 ng) and TAK-242 (75 *μ*g; 7-10 mice per group). After 3 h, mice were sacrificed and articular lavages were performed. (c) Male C57BL/6J wildtype (WT) and TLR4 knockout (TLR4^−/−^) mice were injected i.a. in the knee with 0.9% NaCl (Co) or mu rSAA3 (30 ng; 3-5 mice per group). After 3 h, mice were sacrificed and articular lavages were performed. (a–c) Lavages were subjected to total cell count and cytospins were prepared for differential leukocyte counts by 2 individuals independently. Data represent the total number of neutrophils/ml lavage ± SEM and are derived from 1 (c), 2 (b), or 3 (a) experiment(s). Statistically significant recruitment of neutrophils compared to control mice, determined by the Mann-Whitney *U* test, is indicated by asterisks (^∗^*p* < 0.05; ^∗∗^*p* < 0.01; ^∗∗∗^*p* < 0.001). Statistically significant inhibition of neutrophil recruitment compared to mice injected with mu rSAA3 alone (a and b) or to wildtype mice injected with mu rSAA3 (c), determined by the Mann-Whitney *U* test, is indicated by a dagger (^†^*p* < 0.05).

Other researchers showed that mu rSAA3 stimulated the migration of macrophages via interaction with TLR4 [11, 12]. Therefore, we investigated whether the chemotactic activity of mu rSAA3 (MyBiosource) on neutrophils was also mediated through TLR4. When the TLR4 inhibitor TAK-242 was injected i.p. in mice together with mu rSAA3 (10 ng), the recruitment of neutrophils towards the peritoneal cavity diminished significantly from 22.06 ± 2.76 × 10^4^ neutrophils/ml (*n* = 11; *p* < 0.001 compared to PBS-injected mice) to 12.77 ± 2.33 × 10^4^ neutrophils/ml (*n* = 12; *p* < 0.05) (Figure 5(a)). Injection of TAK-242 alone or of vehicle (0.002% DMSO) did not influence neutrophil migration (data not shown). Similarly, TAK-242 significantly inhibited the influx of neutrophils into the knee cavity when injected together with mu rSAA3 (30 ng) from 10,689 ± 2219 neutrophils/ml in mu rSAA3-injected mice (*n* = 9; *p* < 0.01 compared to control mice) to 4020 ± 1157 neutrophils/ml in mice injected with a combination of mu rSAA3 and TAK-242 (*n* = 10; *p* < 0.05) (Figure 5(b)). It must be noted that we injected TAK-242 together with mu SAA3. Most probably, the efficiency of inhibition might be increased by injecting the antagonist some time before injecting mu SAA3. Upon i.a. injection of mu rSAA3 (30 ng) in TLR4 knockout mice, the recruitment of neutrophils was even reduced to 4350 ± 1195 neutrophils/ml (*n* = 4; *p* < 0.05 compared to wildtype mice injected with mu rSAA3), which was as low as the number of neutrophils recruited to the knee of control TLR4 knockout mice (4491 ± 1836 neutrophils/ml; *n* = 3) (Figure 5(c)). Similarly, mu rSAA3 (10 ng) recruited a statistically significant number of neutrophils towards the peritoneal cavity of wildtype mice, compared to PBS-injected mice (*p* < 0.05). In contrast, the same dose of mu rSAA3 did not stimulate neutrophil migration towards the peritoneal cavity of TLR4 knockout mice upon i.p. injection (Supplemental Figure 1).

### 3.5. Contaminating LPS Potently Induces Chemokines and May Affect the Biological Activity of Mu rSAA3

**Figure 6:**
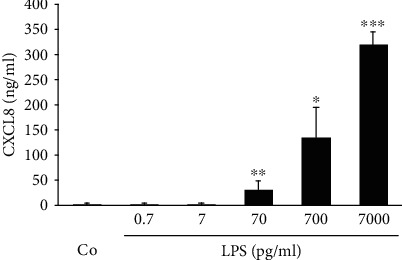
Low concentrations of LPS induce CXCL8 in CD14^+^ monocytes. Human CD14^+^ monocytes derived from buffy coats from healthy donors were stimulated with LPS (0.7-7000 pg/ml) or were left untreated (Co). After 24 h, supernatants were collected and levels of CXCL8 were determined via a specific ELISA. Data represent the mean production of CXCL8 ± SEM derived from 3 to 11 independent experiments. Statistically significant differences from untreated control cells, determined by the Mann-Whitney *U* test, are indicated by asterisks (^∗^*p* < 0.05; ^∗∗^*p* < 0.01; ^∗∗∗^*p* < 0.001).

Until now, mu rSAA3 (MyBiosource) provoked a pronounced induction of chemokines, to an equal degree as LPS, and strongly stimulated the migration of neutrophils. However, the involvement of TLR4 in mu rSAA3-induced neutrophil chemotaxis and the evidence of hu rSAA1 binding to LPS [35] raised some questions. Therefore, mu rSAA3 (MyBiosource) was subjected to an endotoxin test. Although the datasheet from the company stated that the endotoxin content of the preparation was <1 EU/*μ*g of protein and although mu rSAA3 was kept under sterile conditions, we measured an endotoxin content of 2.83 EU/*μ*g mu rSAA3, corresponding to 283-1132 pg LPS/*μ*g protein (Table 1). To determine whether such a small amount of LPS could interfere with the aforementioned biological assays, human CD14^+^ monocytes were induced with low concentrations of LPS for 24 h, whereafter CXCL8 levels were measured in supernatants via a specific ELISA (Figure 6). LPS dose-dependently stimulated the production of chemokine in the cells, with statistically significant amounts of CXCL8 being produced from 70 pg/ml of LPS onwards. These results clearly show that even low concentrations of LPS can evoke significant biological effects.

### 3.6. Purification of Commercial Mu rSAA3 to Homogeneity via RP-HPLC

Knowing that our mu rSAA3 preparation (MyBiosource) was contaminated with LPS and that even small amounts of LPS can cause significant biological effects, we purified mu rSAA3 to homogeneity (mu rSAA3_pur_) using RP-HPLC. Therefore, we purchased mu rSAA3 from another commercial source (Gentaur), which contained less LPS (Table 1). Upon RP-HPLC, the protein eluted from the column in a peak between fractions 52 and 60, corresponding to 42-48% acetonitrile (data not shown). By thoroughly purifying mu rSAA3 by RP-HPLC, we removed bacterial contaminants that could possibly interfere with our biological experiments. This was confirmed through an endotoxin test, which demonstrated that the endotoxin content of mu rSAA3_pur_ was below the detection limit, i.e. <0.005 EU/*μ*g protein, corresponding to <0.5-2 pg LPS/*μ*g mu rSAA3_pur_. In contrast, before purification, mu rSAA3 (Gentaur) contained 0.38 EU endotoxin/*μ*g protein, corresponding to 37-148 pg LPS/*μ*g mu rSAA3 (Table 1).

### 3.7. RP-HPLC-Purified Mu rSAA3 (Mu rSAA3_pur_) Lacks Chemokine-Inducing and Neutrophil Chemotactic Activity

**Figure 7:**
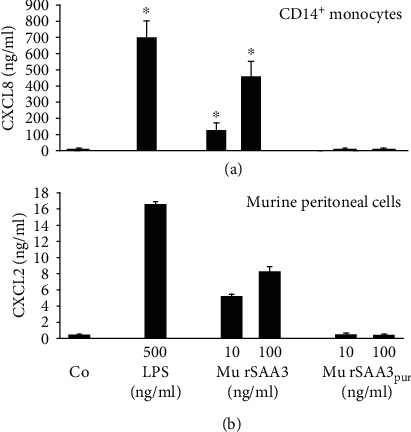
In contrast to impure mu rSAA3, RP-HPLC-purified mu rSAA3 (mu rSAA3_pur_) does not induce chemokines in leukocytes. Human CD14^+^ monocytes derived from buffy coats from healthy individuals (a) or murine peritoneal cells derived from healthy mice (b) were stimulated with LPS (500 ng/ml), mu rSAA3 (10 or 100 ng/ml), mu rSAA3_pur_ (10 or 100 ng/ml) or were left untreated (Co). After 24 h, supernatants were collected and levels of CXCL8 (a) or CXCL2 (b) were determined via specific ELISAs. Data represent the mean production of chemokine ± SEM derived from 4 (a) or 3 (b) independent experiments. Statistically significant differences from untreated control cells, determined by the Mann-Whitney *U* test, are indicated by asterisks (^∗^*p* < 0.05).

**Figure 8:**
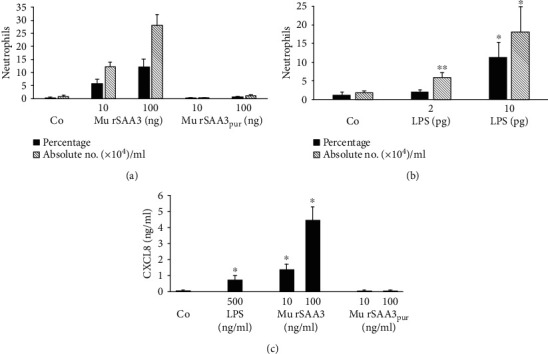
In contrast to impure mu rSAA3, RP-HPLC-purified mu rSAA3 (mu rSAA3_pur_) does not chemoattract neutrophils *in vivo*. (a) Female NMRI mice were injected i.p. with 100 *μ*l of PBS (Co), mu rSAA3 (10 or 100 ng) or RP-HPLC-purified mu rSAA3_pur_ (10 or 100 ng; 3-5 mice per group). After 2 h, mice were sacrificed and peritoneal lavages were performed. Total cell counts in peritoneal lavages were determined and the percentage of neutrophils (Ly-6G^+^CD11b^+^) was quantified via flow cytometry. (b) Female NMRI mice were injected i.p. with 100 *μ*l of PBS (Co) or LPS (2 or 10 pg; 4-8 mice per group). After 2 h, mice were sacrificed and peritoneal lavages were performed. Total cell counts in peritoneal lavages were determined, and cytospins were prepared for differential leukocyte counts by 2 individuals independently. (a and b) Data represent the mean percentage (black bars) or absolute number × 10^4^/ml (hatched bars) of neutrophils ± SEM derived from 1 (a) or 1-3 (b) independent experiment(s). Statistically significant recruitment of neutrophils compared to PBS-injected mice, determined by the Mann-Whitney *U* test, is indicated by asterisks (^∗^*p* < 0.05; ^∗∗^*p* < 0.01). (c) Human CD14^+^ monocytes derived from buffy coats from healthy individuals were stimulated with LPS (500 ng/ml), mu rSAA3 (10 or 100 ng/ml), mu rSAA3_pur_ (10 or 100 ng/ml) or were left untreated (Co). After 2 h, supernatants were collected and levels of CXCL8 were determined via a specific ELISA. Data represent the mean production of chemokine ± SEM derived from 4 independent experiments. Statistically significant differences from untreated control cells, determined by the Mann-Whitney *U* test, are indicated by asterisks (^∗^*p* < 0.05).

After purification via RP-HPLC, the chemokine-inducing capacity of mu rSAA3_pur_ (Gentaur) was tested in parallel with that of impure mu rSAA3 on human CD14^+^ monocytes and murine peritoneal cells (Figure 7). Similar to mu rSAA3 from MyBiosource (Figures 2(a) and 3(c)), mu rSAA3 from Gentaur induced high amounts of chemokines upon 24 h stimulation of these cells, reaching a maximal production of 460 ± 94 ng/ml of CXCL8 in CD14^+^ monocytes (*n* = 4; *p* < 0.05; Figure 7(a)) and 8.3 ± 0.6 ng/ml of CXCL2 in murine peritoneal cells (*n* = 3; Figure 7(b)) at 100 ng/ml. However, upon purification, mu rSAA3_pur_ (Gentaur) completely lost its potency to induce chemokines. The same effect was seen in L929 murine fibroblasts. L929 cells stimulated for 24 h with mu rSAA3_pur_ (40 ng/ml) produced 65.29 ± 6.66 pg/ml CCL2, whereas CCL2 production of untreated cells was 53.22 ± 4.53 pg/ml (*n* = 2). In comparison, cells stimulated with impure mu rSAA3 (40 ng/ml) produced 455.97 ± 11.43 pg/ml CCL2 (*n* = 2). Besides the chemokine-inducing capacity, the *in vivo* neutrophil chemotactic activity of mu rSAA3 (Gentaur) was also lost upon RP-HPLC purification (Figure 8(a)). Mu rSAA3 clearly recruited neutrophils to the peritoneal cavity after i.p. injection of 10 ng (5.8 ± 1.7% neutrophils, corresponding to 12.10 ± 1.90 × 10^4^ neutrophils/ml; *n* = 3) or 100 ng (12.3 ± 2.6% neutrophils, corresponding to 28.05 ± 4.16 × 10^4^ neutrophils/ml; *n* = 3) of the protein, similarly as observed upon injection of mu rSAA3 from MyBiosource (Figure 4(c)). However, neutrophil recruitment completely disappeared upon purification of mu rSAA3 to homogeneity. Indeed, 100 ng of mu rSAA3_pur_ chemoattracted a maximum of 1.19 ± 0.28 × 10^4^ neutrophils/ml peritoneal lavage (0.6 ± 0.1% neutrophils; *n* = 5), which was comparable to the neutrophil recruitment to the peritoneal cavity in PBS-injected control mice (0.65 ± 0.50 × 10^4^ neutrophils/ml; *n* = 4) (Figure 8(a)).

Next, we evaluated whether the *in vivo* neutrophil chemotactic activity of mu rSAA3 (Gentaur) could be explained by the presence of LPS in the preparation. Extrapolation of the results from the endotoxin test (Table 1) revealed that 10 and 100 ng of mu rSAA3 (Gentaur) also contained 0.37-1.48 pg and 3.7-14.8 pg LPS, respectively. Therefore, mice were injected i.p. with 2 or 10 pg LPS and the migration of neutrophils towards the peritoneal cavity was measured 2 h after injection via differential cell count of cytospins (Figure 8(b)). Injection of both doses of LPS provoked a statistically significant recruitment of neutrophils; 5.84 ± 1.34 × 10^4^ (*n* = 8; *p* < 0.01) and 18.20 ± 6.77 × 10^4^ neutrophils/ml (*n* = 4; *p* < 0.05) were present in the peritoneal lavages from mice injected with 2 and 10 pg LPS, respectively. Since these numbers were similar as those observed when mice were injected with 10 or 100 pg mu rSAA3, we can assume that the *in vivo* neutrophil chemotactic activity of impure mu rSAA3 (Figure 8(a)) was indeed provoked by contaminating LPS. It is expected that such low amounts of LPS can induce substantial amounts of chemokines within 2 h after injection of mu rSAA3 explaining its neutrophil chemotactic activity. Indeed, during *in vitro* induction experiments, mu rSAA3 (Gentaur) induced significant amounts of CXCL8 in human CD14^+^ monocytes after 2 h of stimulation (Figure 8(c)). A maximal production of 4.421 ± 0.848 ng/ml of CXCL8 (*n* = 4; *p* < 0.05) was reached upon treatment of monocytes with 100 ng/ml of mu rSAA3.

### 3.8. Mu rSAA3_pur_ Synergizes with CXCL8 in Neutrophil Activation

**Figure 9:**
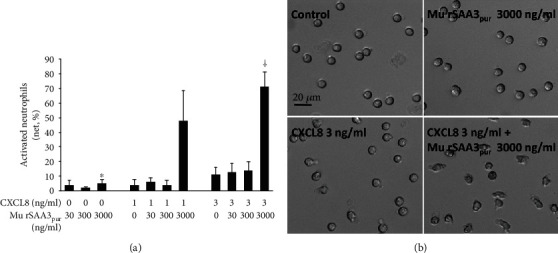
Mu rSAA3_pur_ synergizes with CXCL8 in neutrophil activation. Activation of human neutrophils was assessed via shape change assays. Neutrophils were stimulated for a period of 3 min with different concentrations of CXCL8 (1 or 3 ng/ml), mu rSAA3_pur_ (30-3000 ng/ml) or were left untreated. After fixation of cells, non-activated resting (round) and activated (blebbed and elongated) neutrophils were counted microscopically by 2 individuals independently. (a) Data represent the mean net percentage of activated neutrophils ± SEM derived from 4 independent experiments. Statistically significant activation of neutrophils (compared to controls) and statistically significant synergy (compared to the sum of the values when both agonists are tested separately), determined by the Mann-Whitney *U* test, are indicated by asterisks (^∗^*p* < 0.05) and daggers (^†^*p* < 0.05), respectively. (b) Phase contrast image (20x magnification; scale bar: 20 *μ*m) of one representative experiment illustrating the morphological change of neutrophils following stimulation with buffer (control), CXCL8 (3 ng/ml), mu rSAA3_pur_ (3000 ng/ml) or a combination of CXCL8 and mu rSAA3_pur_ are shown.

In the past, we demonstrated that hu rSAA1 activates (shape change assay) and chemoattracts (Boyden Microchamber Assay) neutrophils in synergy with CXCL8 [26]. We investigated whether mu rSAA3_pur_ (Gentaur) was also able to induce morphological changes in neutrophils by performing shape change assays (Figures 9(a) and 9(b)). Stimulation of neutrophils with 3000 ng/ml of mu rSAA3_pur_ resulted in a low but statistically significant percentage of activated neutrophils (5 ± 3%; *n* = 4; *p* < 0.05). Treatment of the cells with a combination of mu rSAA3_pur_ (3000 ng/ml) and CXCL8 (3 ng/ml) further increased the percentage of activated neutrophils to 72 ± 10%, which is statistically significantly higher than the sum of the percentage of activated neutrophils upon stimulation of cells with mu rSAA3_pur_ (3000 ng/ml) or CXCL8 (3 ng/ml; 11 ± 5% activated neutrophils) alone (*n* = 4; *p* < 0.05).

## 4. Discussion

Since SAA is highly conserved throughout evolution, one must think that an exclusive and indispensable function is granted to this acute phase protein. During the past decades, the role of SAA has been broadly investigated and a wide variety of functions has been attributed to SAA [8]. However, most of the research was done with SAA recombinantly expressed in *E. coli*. This last fact was very often overlooked, since companies supplying this recombinant SAA assured that the endotoxin content of these preparations was minimal. Moreover, researchers performed extra LPS tests and also included controls in their experiments to ensure that results were truly caused by SAA itself and not by contaminating products. However, bacterial contamination does not only include LPS, acting through TLR4, but also lipoproteins and formylated peptides, exerting their biological effects through activation of TLR2 and FPR1/2, respectively. Coincidence or not, TLR2, TLR4, and FPR2 are also the receptors responsible for several biological activities of SAA following several publications [33, 36–47]. Nevertheless, only very recently the role of contaminating bacterial products in commercially available recombinant SAA was evidenced. Indeed, Burgess *et al*. revealed that commercial SAA1 produced in *E. coli* contained numerous bacterial proteins, including lipoproteins, and that these lipoproteins and not SAA itself are responsible for the TLR2-mediated induction of cytokines in cells. Recombinant SAA1 derived from eukaryotic cells did not induce such an effect, but did so after adding lipoproteins to the preparation [48]. Almost simultaneously, Cheng *et al.* showed that recombinant SAA1 is able to bind LPS in a dose-dependent manner, diminishing LPS-induced proinflammatory effects [35]. In fact, SAA1 was shown to bind a variety of ligands, including lysophospholipids [49, 50], HDL and other lipoproteins [51, 52], cholesterol [53] and retinol [54, 55].

In this study, we show that commercially available murine SAA3, recombinantly expressed in *E. coli* (mu rSAA3), is a potent inducer of chemokines *in vitro* (Figures 2 and 3) and a strong chemoattractant *in vivo* (Figure 4(c)). However, the same SAA3, purified to homogeneity by RP-HPLC (mu rSAA3_pur_), lacks these inflammatory activities (Figures 7 and 8). This indicates that further purification by RP-HPLC of mu rSAA3 implied complete removal of interfering bacterial products. However, the synergy between mu rSAA3 and CXCL8 in neutrophil shape change was retained when using mu rSAA3_pur_ (Figures 9(a) and 9(b)). In fact, these findings remind us of the differences in inflammatory activities observed between endogenous and recombinant SAA1, reported several years ago. In these reports, it was shown that, in contrast to recombinant SAA1, endogenous SAA1 did not induce cytokines (e.g. CXCL8) in monocytes or neutrophils and did not activate neutrophils, assessed by measuring the shedding of L-selectin [56, 57].

The fact that the *in vivo* recruitment of neutrophils towards mu rSAA3 was mediated by TLR4 (Figure 5) and that commercial recombinant SAA1 is contaminated with lipoproteins and is effectively able to bind LPS [35, 48], raised some concern. Although the datasheet of mu rSAA3 from MyBiosource mentioned an endotoxin level of <1.0 EU LPS/*μ*g protein, our endotoxin tests indicated that the endotoxin content was 2.83 EU/*μ*g protein, corresponding to 283-1132 pg LPS/*μ*g protein, which exceeds the endotoxin level stated by the company by approximately threefold. Similarly, Schwarz *et al.* tested the endotoxin content of several commercially available recombinant proteins and found that the LPS contamination levels in these protein preparations were sometimes higher than the maximal level stated in the data sheets [58]. Extrapolation of the results from our endotoxin tests to the induction experiments performed with mu rSAA3 reveals that CD14^+^ monocytes stimulated with 1000 ng/ml of mu rSAA3 (Figure 2) were in fact also subjected to 283 pg/ml of LPS. In our hands, treatment of CD14^+^ monocytes with LPS at a dose as low as 70 pg/ml elicited a statistically significant production of CXCL8 by the cells. By increasing the concentration of LPS up to 7000 pg/ml, CXCL8 production levels of about 300 ng/ml were reached after 24 h induction (Figure 6). The mu rSAA3 preparation from Gentaur elicited a clear influx of neutrophils upon i.p. injection of 10 or 100 ng of protein (Figure 8(a)). Although the LPS content of this preparation was within the limits stated by the company (0.38 EU LPS/*μ*g protein), the amount of endotoxin remaining in the preparation was still able to provoke a statistically significant influx of neutrophils towards the peritoneal cavity (Figure 8(b)), equivalent to that observed after injection of mu rSAA3. Indeed, 10 or 100 ng of mu rSAA3 also contained 0.4-1.5 pg or 4-15 pg LPS, respectively. Apart from LPS, also other bacterial contaminants, such as lipoproteins [48] and formylated peptides, could be present in mu rSAA3. Besides the possible inefficiency of the antagonist when injected together with mu rSAA3, the partial inhibition of *in vivo* recruitment of neutrophils towards mu rSAA3 by TAK-242 could also be due to the presence of bacterial contaminants other than LPS in the mu rSAA3 preparation. PGN, a component of the cell wall of Gram-positive and Gram-negative bacteria activating TLR2 [59], induced statistically significant amounts of chemokine in murine macrophages from a concentration of 1 ng/ml onwards. Moreover, similar levels of chemokine were produced by mu rSAA3-stimulated cells as that observed with PGN (Figure 3(a)). Overall, this means that contamination of reagents with even very low concentrations of bacterial products can dramatically influence experimental results. The inability of mu rSAA3_pur_ to exert any biological effect cannot be caused by the toxicity of reagents used during the purification process, since mu rSAA3_pur_ synergized with CXCL8 in neutrophil activation experiments (Figures 9(a) and 9(b)). Likewise, chemically synthesized fragments of human SAA1, which were purified via RP-HPLC, also synergized with chemokines in leukocyte activation and migration [60, 61].

The receptor usage of murine SAA3 to exert its effects has until now been studied less than that of human SAA1. The receptor proposed for SAA3-mediated induction of cytokines is TLR4 [12, 21]. For instance, mu rSAA3 expressed in *E. coli* induced TNF-*α* and IL-6 mRNA in CMT-93 epithelial cells. Treatment of the cells with the TLR4 inhibitor TAK-242 prior to stimulation with mu rSAA3 diminished cytokine expression [21]. However, most of the experiments investigating the receptor usage of mu rSAA3 were executed with impure mu rSAA3, which probably contains enough LPS to exert the described TLR4-mediated effects of mu rSAA3. With regard to chemotaxis, full-length *E. coli*-derived mu rSAA3, treated with polymixin B, stimulated *in vitro* migration of peritoneal macrophages from wildtype mice, whereas this was not the case when the cells were derived from TLR4 knockout mice. In addition, mammalian cell-derived mu rSAA3 was found to be chemotactic for RAW264.7 macrophages and LLC epithelial cells to a similar extent as *E. coli*-derived mu rSAA3 [11, 12]. Moreover, synthetic mu SAA3 (1-67), which is not from bacterial origin and which contained LPS levels referred to as “endotoxin-free,” was shown to be chemotactic for peritoneal macrophages through binding to MD-2 of the TLR4/MD-2 complex [11]. In our hands, purified mu SAA3 could not induce neutrophil recruitment in wildtype mice. We, therefore, speculate that FPR2 might be the functional receptor for mu SAA3, similar to hu SAA1. Besides bacterial formylated peptides, FPR2 also interacts with a variety of ligands, including SAA1 [36]. The chemotactic activity of hu rSAA1 is mediated by FPR2, since this protein induced an increase in intracellular calcium concentration in FPR2-transfected HEK293 cells and stimulated the migration of these cells [37, 62]. In line with these findings, we demonstrated that the synergy between hu rSAA1 and CXCL8 in neutrophil chemotaxis was mediated by binding to FPR2, and that hu rSAA1, purified to homogeneity via RP-HPLC, did not display any TLR-mediated biological effects but retained its potential to activate FPR2 [26, 63]. The homology in amino acid sequence between murine SAA3 and SAA1 is 70%, which favors the plausibility of a shared receptor between both SAA variants.

In the past, other researchers have reported a distortion of experimental results caused by bacterial contamination of recombinant proteins expressed in *E. coli* and have already warned about their use [64]. Kringle 5, derived from plasminogen, was previously described to inhibit endothelial cell growth. However, after purification via RP-HPLC, this inhibition was completely lost [65]. Furthermore, the massive induction of the acute phase response after intravenous injection of human subjects with recombinant C-reactive protein (CRP) [66], which is together with SAA one of the major acute phase proteins in humans, appeared to be evoked by contaminating bacterial products [67]. The authors measured an endotoxin level of 46.6 EU/mg of recombinant protein, which is about 60-fold less than the endotoxin level observed in mu rSAA3 (MyBiosource). They showed that the clear proinflammatory effects provoked by recombinant CRP were not at all present when endogenous CRP was used.

In conclusion, when using recombinant proteins originating from bacteria, a proper quality control before performing experiments is highly recommended. Experimental results obtained by using these recombinant proteins should always be interpreted with care and it would be better to use nonbacterial expression systems, such as eukaryotic cells, or transgenic or knockout mice in the future. Alternatively, purification of recombinant proteins through in-depth chromatography can also be considered to eliminate any bacterial contamination.

## 5. Conclusions

Taken together, we make the following conclusions:

1. Most if not all Toll-like receptor-mediated activities ascribed to mu SAA3 are due to contamination with lipopolysaccharides and/or lipoproteins, including its potential to induce inflammatory mediators such as cytokines and chemokines
2. In-depth purification of recombinant proteins to obtain a homogeneous product is required and avoids false positive results as published before for mu SAA3
3. Most probably, SAA can still be classified as a mediator of inflammation, because the homogeneous protein retains its capacity to synergize with chemokines in leukocyte recruitment through the G protein-coupled receptor FPR2

## Figures and Tables

**Table 1:** Endotoxin level in impure and RP-HPLC-purified mu rSAA3 and hu rSAA1.

Reagent	Company	Endotoxin level^a^ (EU/*μ*g of protein)			Conversion^b^ to pg/*μ*g of protein	
		Data sheet company	Before RP-HPLC	After RP-HPLC	Before RP-HPLC	After RP-HPLC
Mu rSAA3	MyBiosource	<1	2.83	ND^c^	283-1132	ND
Mu rSAA3	Gentaur	<1	0.38	<0.005	37-148	<0.5-2
Hu rSAA1	Peprotech	<1	0.0029	ND	0.3-1.2	ND

^a^The endotoxin level was measured via the LAL assay. ^b^Assuming that 1 EU/ml corresponds to 0.1-0.4 ng of endotoxin/ml. ^c^Not determined.

## Data Availability

The datasets generated during and/or analyzed during the current study are available from the corresponding author upon reasonable request.

## Supplementary Materials

Supplementary Materials Figure 1: In vivo recruitment of neutrophils towards impure mu rSAA3 is mediated by activation of TLR4. Male C57BL/6J wildtype (WT) and TLR4 knockout (TLR4-/-) mice were injected i.p. with PBS (control) or mu rSAA3 (10 ng; 4 mice per group). After 2 h, mice were sacrificed and peritoneal lavages were performed. Lavages were subjected to total cell count, and cytospins were prepared for differential leukocyte counts. Data represent the total number of neutrophils/ml lavage ± SEM and are derived from 1 experiment. Statistically significant recruitment of neutrophils compared to control mice, determined by the Mann-Whitney *U* test, is indicated by asterisks (^∗^*p* < 0.05).
