# Supplementary material for: Biological Characterization of Commercial Recombinantly Expressed Immunomodulating Proteins Contaminated with Bacterial Products in the Year 2020: The SAA3 Case
Source: Mediators Inflamm. 2020 Jul 6;2020:6087109. doi: 10.1155/2020/6087109 (PMC7362292; doi:10.1155/2020/6087109)
Supplement: Supplementary Materials — Figure 1: In vivo recruitment of neutrophils towards impure mu rSAA3 is mediated by activation of TLR4. Male C57BL/6J wildtype (WT) and TLR4 knockout (TLR4-/-) mice were injected i.p. with PBS (control) or mu rSAA3 (10 ng; 4 mice per group). After 2 h, mice were sacrificed and peritoneal lavages were performed. Lavages were subjected to total cell count, and cytospins were prepared for differential leukocyte counts. Data represent the total number of neutrophils/ml lavage ± SEM and are derived from 1 experiment. Statistically significant recruitment of neutrophils compared to control mice, determined by the Mann-Whitney U test, is indicated by asterisks (∗p < 0.05). [file 6087109.f1.pdf]

Supplemental Figures

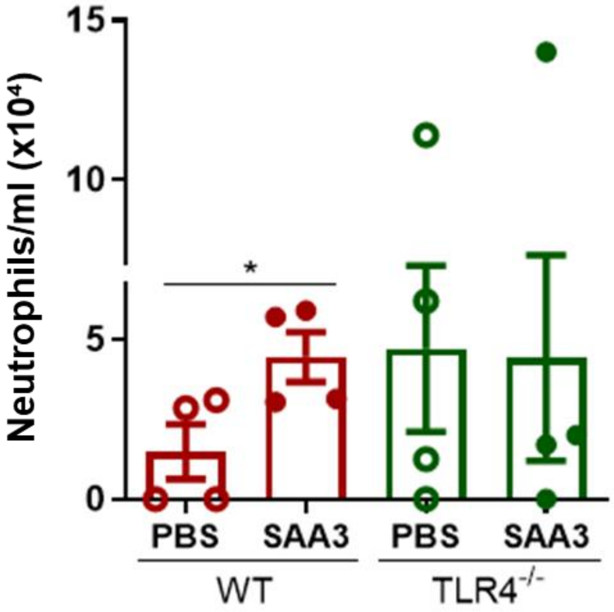

Figure 1. *In vivo* recruitment of neutrophils towards impure mu rSAA3 is mediated by activation of TLR4. Male C57BL/6J wildtype (WT) and TLR4 knockout (TLR4$^{-/-}$) mice were injected i.p. with PBS (control) or mu rSAA3 (10 ng; 4 mice per group). After 2 h, mice were sacrificed and peritoneal lavages were performed. Lavages were subjected to total cell count and cytospins were prepared for differential leukocyte counts. Data represent the total number of neutrophils/ml lavage ± SEM and are derived from 1 experiment. Statistically significant recruitment of neutrophils compared to control mice, determined by the Mann-Whitney U test, is indicated by asterisks (*p<0.05).
